# Analysis of the Association between Telomere Length and Neurological Disability in Stroke Types

**DOI:** 10.3390/medicina60101657

**Published:** 2024-10-10

**Authors:** Sang-Hun Lee, Tae-Kwon Kim, Jong-Hoon Yoo, Hyung-Jong Park, Jae-Hyun Kim, Jae-Ho Lee

**Affiliations:** 1 Department of Emergency Medicine, Keimyung University Dongsan Hospital, Keimyung University School of Medicine, Daegu 42601, Republic of Korea; kimtaegoi@dsmc.or.kr (T.-K.K.); 210035@dsmc.or.kr (J.-H.Y.); 2 Department of Neurology, Keimyung University Dongsan Hospital, Keimyung University School of Medicine, Daegu 42601, Republic of Korea; phj209042@dsmc.or.kr; 3 Department of Neurosurgery, Keimyung University Dongsan Hospital, Keimyung University School of Medicine, Daegu 42601, Republic of Korea; kdhggo@dsmc.or.kr; 4 Department of Anatomy, Keimyung University School of Medicine, Daegu 42601, Republic of Korea; anato82@dsmc.or.kr

**Keywords:** acute ischemic stroke, telomere, emergencies

## Abstract

*Background and Objectives*: The association between neurological disability, prognosis, and telomere length (TL) in patients with stroke has been investigated in various ways. However, analysis of the type of stroke and ischemic stroke subgroups is limited. In this study, we aimed to determine the association between TL and neurological disability according to stroke type. *Materials and Methods*: This prospective study included patients with stroke who visited a single-center emergency department (ED) between January 2022 and December 2023. The association between TL and neurological disabilities, using the Modified Rankin Scale (mRS) and National Institutes of Health Stroke Scale (NIHSS), was evaluated according to the patient’s stroke type and subgroup of ischemic stroke. Multivariate analysis was performed to determine the association between neurological disabilities in patients with ischemic stroke and the subgroups. *Results*: A total of 271 patients with stroke were enrolled. The NIHSS score was found to be higher at the time of ED visit (adjusted odds ratio [OR], 5.23; 95% confidence interval [CI], 1.59–17.2, *p* < 0.01) and 1 day later (adjusted OR, 7.78; 95% CI, 1.97–30.70, *p* < 0.01) in the ischemic stroke group with a short TL. In the other determined etiology (OD) or undetermined etiology (UD) group, the NIHSS was higher in the short TL group at the ED visit (adjusted OR, 7.89; 95% CI, 1.32–47.25, *p* = 0.02) and 1 day after (adjusted OR, 7.02; 95% CI, 1.14–43.47, *p* = 0.04). *Conclusions*: TL is associated with neurological disability in early ischemic stroke and is prominent in the UD and OD subgroups.

## 1. Introduction

Telomeres are nuclear protein complexes located at the ends of chromosomes that protect chromosomes against degradation and maintain chromosome integrity [1]. Telomeres are shortened in each cell cycle and are associated with general human aging and age-related diseases [2].

Declines in telomere length (TL) are affected by several factors, including age. Moreover, the shortening of TL is affected by lifestyle factors, such as smoking, drinking, and physical activity, as well as by differences between men and women [3]. In addition, TL is associated with various diseases such as cancer, heart disease, lung disease, liver disease, and hematologic diseases [4,5]. TL is associated not only with chronic diseases but also with acute-stage diseases such as sepsis, myocardial infarction, and acute ischemic stroke [6,7,8].

Stroke can be classified as ischemic stroke, hemorrhagic stroke, or transient ischemic attack (TIA). Ischemic stroke is classified into five subtypes by the Trial of Org 10,172 in acute stroke treatment (TOAST): large-artery atherosclerosis (LAA); >50% stenosis or occlusion of an extracranial or intracranial major vessel or branch artery, as shown in a duplex or angiographic image, for small-vessel occlusion (SVO); computed tomography/magnetic resonance imaging showing white matter lesions and/or infarction < 1.5 cm in diameter or cardioembolism (CE); a major cardioembolic source is detected, or stroke of other determined etiology (OD); other causes are identified like vasculitis, hypercoagulable state or hematologic disorder, and stroke of undetermined etiology; and two or more causes are identified, negative evaluation, or incomplete evaluation (UD) [9]. Despite various classifications of these strokes, previous studies have only analyzed the association between ischemic stroke and TL. Therefore, we analyzed the relationship between TL and neurological disability in patients with stroke, including those with hemorrhagic stroke, TIA, and an ischemic stroke subtype.

## 2. Materials and Methods

### 2.1. Study Design and Search Strategy

This single-center prospective study was performed at Keimyung University Dongsan Hospital, South Korea, between January 2022 and December 2023. This study was conducted only on adult patients (≥18 years old) diagnosed with stroke in the emergency department (ED). Patients within 24 h of developing neurological symptoms were enrolled, and the standard of time was the last normal time (LNT). Patients whose basic character identification of the patient was insufficient or whose neurological examination findings were omitted were excluded as missing data. Patients who visited the ED and performed only conservative treatment without wanting active treatment, those who were transferred, or those who returned home were excluded. Patients who had already been diagnosed at another hospital and were transferred to the hospital were excluded. Patients were also excluded when the patient or guardian did not want additional blood collection or to sign consent. Patients were followed up for 7 days after hospital admission or at the time of death. The patients were categorized into shorter and longer TL groups based on the lowest quarter of the TL of patients with ischemic stroke. The study protocol was approved by the Institutional Review Board (IRB number: 2021-11-022-007), and informed consent was obtained before patient enrollment.

Data on patient characteristics such as age, sex, height, weight, smoking, alcohol consumption, previous illness, mental status, vital signs, and time taken for management were collected. Laboratory results were also collected, including white blood cells, C-reactive protein, lactic acid, hemoglobin, creatinine, albumin, total cholesterol, high-density lipoprotein cholesterol, low-density lipoprotein cholesterol, triglyceride, glucose, HbA1C, apolipoprotein A1, and apolipoprotein B. Neurological evaluation was divided into seven stages from 0 to 6 using the Modified Rankin Scale (mRS) [10]. The mRS was collected before and after the stroke, and the difference between the two values was recorded. Another neurological evaluation, the National Institutes of Health Stroke Scale (NIHSS), was performed on patients with ischemic stroke [11]. This score was obtained at the time of the ED visit and after 1 day and 7 days. In patients with a transient ischemic attack (TIA), the ABCD2 score was calculated [12]. The patient’s diagnosis and management were finally made by experts in the emergency medicine, neurology, and neurosurgery departments.

TL blood samples were obtained from approximately 1 mL of blood collected previously from the patient and stored in heparin-treated tubes. DNA was extracted from the blood samples using a QIAamp DNA Mini Kit (Qiagen, Inc., Valencia, CA, USA). Telomere length was measured using qPCR. The target primer used telomere (T) and β-globin (B). The telomere length was expressed by the relative telomere value, which was obtained through the following formula: relative telomere length (T/B) = 2 − ΔCq, where ΔCq = mean CqT − mean CqS.

### 2.2. Statistical Analysis

Continuous variables were expressed as medians and quartiles. The two groups were compared using Student’s *t*-test for normally distributed variables and the Mann–Whitney U test for variables with non-normal distribution. Categorical variables were expressed as numbers and percentages. The two groups were compared using the χ^2^ test or Fisher’s exact test. The associations between neurological disability and TL were analyzed using univariate logistic regression analysis. The patients with neurological disabilities were categorized into two groups. The NIHSS score was either divided by a value greater than 5 or not, and the mRS was divided by a value greater than 2. Variables were adjusted by sex, age, height, weight, smoker, alcohol drinking, hypertension, diabetes, hypercholesterol, coronary artery disease, cerebrovascular accident, atrial fibrillation/flutter, chronic kidney disease, anti-coagulation medication, systolic blood pressure, diastolic blood pressure, heart rate, respiratory rate, body temperature, albumin, total cholesterol, high-density lipoprotein (HDL) cholesterol, low-density lipoprotein (LDL) cholesterol, triglyceride, apolipoprotein A1, and apolipoprotein B, and these were analyzed alongside last normal time to door time by multivariate logistic regression analysis; the results were reported as odds ratio (OR) and 95% confidence interval (CI). We analyzed all types of stroke, including ischemic stroke, hemorrhagic stroke, and transient ischemic attack (TIA), as well as the subgroups of ischemic stroke, such as LAA, SVO, CE, OD, and UD. All statistical analyses were performed using SPSS version 27.0 for Windows (SPSS Inc., Armonk, NY, USA).

## 3. Results

**Figure 1:**
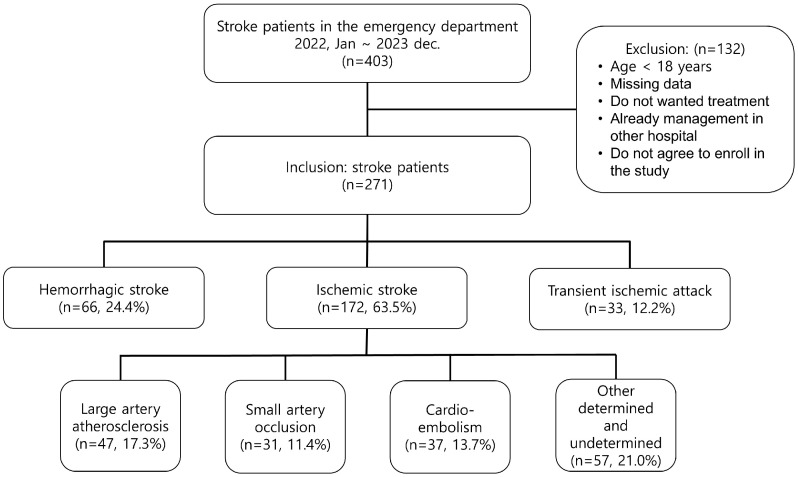
Flowchart of the study patients.

During the study period, 403 patients with acute stroke visited the ED; 271 patients with stroke were finally enrolled after excluding ineligible patients. There were 172 patients with ischemic stroke (63.5%), 66 with hemorrhagic stroke (24.4%), and 33 with TIA (12.2%). In patients with ischemic stroke, who were divided according to the TOAST classification, 47 (17.3%) had LAA, 31 (11.4%) had SVO, 37 (13.7%) had CE, and 57 (21.0%) had OD or UD. (Figure 1).

The mean age of the participants was 70 years, and 149 (55.0%) were male. Among the patients, 184 (67.9%) were alert, 48 (17.7%) were drowsy, 24 (8.9%) were in a stupor, 11 (4.1%) were semi-comatose, and 4 (1.5%) were comatose. In patients with ischemic stroke, door-to-computed tomography (CT) scans took an average of 23 min, door-to-intravenous thrombolysis (IVT) took 38 min, and door-to-endovascular treatment (EVT) took 130 min. The pre-mRS score of the usual state before stroke was 0 points, and the mRS score on arrival at the ED was 3 points. The difference between these two values, delta-mRS, was two points. The NIHSS was five points at the time of ED visit, five points after 1 day, and three points after 7 days. The mean ABCD2 score was four. A total of 243 (89.7%) patients survived until discharge (Table 1).

In all patients with stroke, differences in neurological disability, determined using mRS and NIHSS scores and according to TL, were not statistically significant. In patients with hemorrhagic stroke and TIA, no differences in the mRS score according to TL were observed. However, in the ischemic patients with stroke, there was an association between TL and neurological disability such as delta-mRS (Crude OR, 2.47; 95% CI, 1.09–5.58, *p* = 0.03), NIHSS at ED visit (Crude OR, 2.66; 95% CI, 1.31–5.44, *p* < 0.01), and NIHSS 1 day after (Crude OR, 3.20; 95% CI, 1.54–6.66, *p* < 0.01) (Table 2).

Multivariate analysis showed that NIHSS was found to be higher at the time of the ED visit (adjusted OR, 5.23; 95% CI, 1.59–17.2, *p* < 0.01) and 1 day later (adjusted OR, 7.78; 95% CI, 1.97–30.70, *p* < 0.01) in the group of patients with ischemic stroke with a short TL. (Table 3).

When patients with ischemic stroke were divided into subgroups according to the TOAST classification, LAA, SVO, and CE did not show statistical significance with respect to TL. However, in the OD or UD group, the NIHSS was higher in the short TL group at the ED visit (adjusted OR, 7.89; 95% CI, 1.32–47.25, *p* = 0.02) and 1 day after (adjusted OR, 7.02; 95% CI, 1.14–43.47, *p* = 0.04) (Table 4).

## 4. Discussion

This study analyzed the association between TL and neurological disability in patients with stroke according to the type of stroke and the subgroups of ischemic stroke. We found that the short TL group had poor neurological disability for patients with ischemic stroke. Additionally, in the subgroup of patients with ischemic stroke, short TLs were associated with poor neurological findings in the OD and UD groups.

A reduction in brain tissue resilience in patients with ischemic stroke can be observed through TL, indicating biological aging. In advanced atherosclerosis, the cell turnover is accelerated in aged cells, phagocytic clearance is reduced, and tissue inflammation and matrix degradation occur, resulting in the thinning of the fibrous cap and ultimately increasing the risk of stroke [13,14]. The aging of the vascular smooth muscle, a crucial factor in stroke, can also be observed through shorter TLs, through which the risk of stroke can be evaluated [14]. Chronic stress, such as shear stress, oxidative stress, and inflammation, shortens the TL in cells, leading to worsening atherosclerosis and is associated with the risk of stroke [15]. In a study regarding the relationship between atherosclerosis and a short TL, this was initially not affected; however, atherosclerosis progressed and was associated with plaque filing/rupture and atherothrombosis in the advanced stage [16].

Several studies have been conducted on the risks and relevance of TL in clinical brain diseases. TL is known to reflect brain aging by showing brain atrophy and white matter hyperintensities of the brain [17]. TL serves as an epigenomic marker of Alzheimer’s disease, a representative of degenerative brain disease [18]. The association between TL and the risk of ischemic stroke was also revealed through a meta-analysis [19]. Short TLs have been shown to increase the risk of stroke and cause a higher incidence of stroke owing to CE in patients with atrial fibrillation [20,21]. In addition, prognosis can be predicted through TL in patients with stroke. Even when the size of the infarction, known to have a decisive effect on the neurological disability of patients with ischemic stroke, was analyzed together with other factors, it was found that the NIHSS score was higher at admission in patients with a short TL [8]. In another prognosis, the mortality rate was also found to be high after stroke in patients with a short TL; moreover, cognitive impairment and dementia, which are caused post-stroke, were found to be more common [22,23].

Similar to previous studies, this study investigated the association between short TLs and stroke. In the group of patients with ischemic stroke, poor neurological disability findings were found in patients with stroke and a short TL. When classified as the cause of ischemic stroke according to the TOAST classification, poor neurological disability was found in patients with a short TL in the UD and OD groups. To the best of our knowledge, this is the first study to investigate the association of TL with neurological symptoms according to the subclassification of stroke. The location and size of brain lesions due to stroke affect neurological symptoms at admission and discharge [24]. Neurological disability appears not only in ischemic stroke but also in hemorrhagic stroke, depending on the mechanism and size of the lesion that is triggered [25,26]. However, according to previous studies, even when the size of these brain lesions was analyzed together as a correction factor, short TL showed more serious neurological symptoms in patients with ischemic stroke [8]. However, this study only targeted patients with ischemic stroke and did not analyze patients with hemorrhagic stroke and TIA, and no analysis was performed on the subgroups of patients with ischemic stroke. Our study involved subclassification and showed poor neurological disability in patients with a short TL in the UD and OD groups. When ischemic stroke occurs due to LAA and SVO causes, the range of infarction is greater than that of other causes, and neurological symptoms resulting from this have a very large impact. It is judged that the effect of underlying disease, age, and TL is relatively low because the effect on large occlusion blood vessels is large. However, the range of infarction caused by UD and OD is relatively small, and the neurological disorders that occur are considered to be affected by the overall patient’s general condition. In UD and OD, underlying diseases such as vasculitis, hypercoagulable state, or hematologic disorder cause ischemic stroke, and ischemic stroke occurs due to the deterioration of these diseases. The deterioration of underlying conditions shows a decrease in TL, and the degree of neurological disability is considered to be significant depending on the degree of deterioration caused by underlying disease. In addition, telomere length is shortened by 30–200 bp when somatic cell division is performed, and it is known that somatic cell division occurs for at least several hours. In the case of ischemic stroke caused by the sudden occlusion of large blood vessels, it is judged that there may not be enough time for TL to be short, but in UD and OD, where ischemic stroke occurs due to the continuous deterioration caused by underlying diseases, TL is already expected to be short. Also, the degree of neurological disability when diagnosing ischemic stroke is considered to reflect the deterioration of underlying diseases.

This study has some limitations. First, this study did not analyze the size and location of the brain lesions caused by stroke. Second, owing to the short follow-up period, outcomes such as long-term mortality and long-term neurological prognosis were not analyzed. Third, this study had a relatively small sample size and was conducted at a single center consisting of patients with Korean ethnicity, thereby limiting the generalizability of the results.

## 5. Conclusions

TL can serve as a biomarker that can be used in the initial neurological evaluation of patients with acute stroke, and it is considered to be more useful in UD and OD patients in particular. These results are limited to the initial evaluation of patients with stroke in a single hospital; thus, a study on the association between large-scale and long-term neurological disorders and TL is needed in the future.

## Figures and Tables

**Table 1:** Demographic and clinical characteristics of the patients enrolled in the study.

	Total (n = 271)	Short Telomere Length (n = 61)	Long Telomere Length (n = 210)	*p*-Value
Age (years)	70 (60–79)	71 (61–82)	69 (59–79)	0.208
Sex (Male)	149 (55.0)	24 (39.3)	125 (59.5)	0.004
Height (cm)	163 (155–172)	161 (155–169)	165 (155–173)	0.024
Weight (kg)	61 (55–70)	60 (54–64)	62 (55–70)	0.018
Smoker, n (%)				
Non-smoker	140 (51.7)	35 (57.4)	105 (50.0)	0.492
Ex-smoker	41 (15.1)	4 (6.6)	37 (17.6)	0.034
Smoker	55 (20.3)	11 (18.0)	44 (21.0)	0.053
Alcohol drinker	85 (31.4)	16 (26.2)	69 (32.9)	0.329
Previous illness				
Hypertension	181 (68.3)	38 (62.3)	143 (68.1)	0.243
Diabetes	86 (31.7)	21 (34.4)	65 (31.0)	0.357
Hypercholesterol	56 (20.7)	15 (24.6)	41 (19.5)	0.245
Coronary artery disease	24 (8.9)	6 (9.8)	18 (8.6)	0.464
Cerebrovascular accident	69 (25.5)	14 (23.0)	55 (26.2)	0.371
Atrial fibrillation//flutter	34 (12.5)	6 (9.8)	28 (13.3)	0.315
Chronic kidney disease	17 (6.3)	6 (9.8)	11 (5.2)	0.157
Anti-coagulation medication	90 (33.2)	20 (32.8)	70 (33.3)	0.534
Mental status				
Alert	184 (67.9)	45 (73.8)	139 (66.2)	0.169
Drowsy	48 (17.7)	8 (13.1)	40 (19.0)	0.191
Stupor	24 (8.9)	6 (9.8)	18 (8.6)	0.464
Semi-coma	11 (4.1)	0 (0)	11 (5.2)	0.057
Coma	4 (1.5)	2 (3.3)	2 (1.0)	0.219
Vital sign				
Systolic blood pressure, mmHg	158 (140–175)	158 (140–170)	160 (140–180)	0.295
Diastolic blood pressure, mmHg	90 (80–100)	89 (76–100)	90 (80–100)	0.035
Heart rate, rate/minute	81 (71–91)	80 (71–86)	82 (71–92)	0.138
Respiratory rate, rate/minute	20 (19–20)	20 (19–20)	20 (19–20)	0.328
Body temperature, °C	36.6 (36.4–36.8)	36.6 (36.4–36.8)	36.7 (36.4–36.8)	0.296
Laboratory				
WBC, ×10^3^/µL	7.43 (6.28–9.91)	7.38 (6.38–9.86)	7.53 (6.24–9.98)	0.471
CRP, mg/dL	0.1 (0.1–0.3)	0.1 (0.1–0.3)	0.1 (0.1–0.3)	0.270
Lactic acid, mmol/L	1.6 (1.2–2.2)	1.6 (1.1–2.4)	1.6 (1.2–2.2)	0.422
Hemoglobin, g/dL	13.5 (12.2–14.6)	13.3 (11.2–14)	13.6 (12.2–14.6)	0.048
Creatinine, mg/dL	0.9 (0.7–1.1)	0.8 (0.7–1.1)	0.9 (0.7–1.1)	0.378
Albumin, g/dL	4.2 (3.9–4.5)	4.2 (4.0–4.5)	4.2 (3.9–4.5)	0.236
Total cholesterol, mg/dL	163 (129–190)	166 (128–192)	161 (130–190)	0.278
HDL cholesterol, mg/dL	49 (39–60)	46 (37–59)	49 (39–60)	0.330
LDL cholesterol, mg/dL	97 (69–126)	100 (71–129)	96 (69–126)	0.495
Triglyceride, mg/dL	95 (67–143)	94 (73–129)	95 (66–147)	0.303
Glucose, mg/dL	136 (112–172)	131 (109–150)	140 (113–178)	0.036
HbA1C, %	6.1 (5.7–6.7)	6.1 (5.7–7.0)	6.1 (5.7–6.7)	0.453
Apolipoprotein A1, mg/dL	135 (116–151)	127 (115–147)	136 (116–154)	0.185
Apolipoprotein B, mg/dL	83 (68–108)	85 (70–107)	83 (67–108)	0.384
Time to taken manage				
LNT-to-door time, minute	228 (78–690)	247 (93–694)	217 (76–692)	0.396
Door-to-CT, minute	23 (17–35)	30 (20–42)	23 (16–32)	<0.001
Door-to-IVT, minute	38 (27–44)	32 (26–58)	42 (27–61)	0.193
Door-to-EVT, minute	130 (107–236)	124 (101–142)	135 (110–279)	0.211
mRS				
Pre-mRS	0 (0–1)	0 (0–1)	0 (0–1)	0.360
mRS at ED visit	3 (1–5)	2 (1–5)	3 (1–5)	0.240
Delta mRS	2 (1–4)	1 (0–4)	2 (1–4)	0.137
NIHSS				
NIHSS at ED visit	5 (2–12)	3 (1–13)	6 (2–12)	0.358
1 day after NIHSS	5 (1–11)	3 (1–15)	6 (1–11)	0.325
7 days after NIHSS	3 (0–10)	2 (0–11)	4 (1–10)	0.232
ABCD2 score	4 (3–5)	4 (3–6)	4 (3–5)	0.468
Survival discharge	243 (89.7)	56 (91.8)	187 (89.0)	0.363
Diagnosis				
Acute ischemic stroke	172 (63.5)	43 (70.5)	129 (61.4)	0.126
Large artery atherosclerosis	47 (17.3)	10 (16.4)	37 (17.6)	0.315
Small artery occlusion	31 (11.4)	9 (14.8)	22 (10.5)	0.358
Cardioembolism	37 (13.7)	10 (16.4)	27 (12.9)	0.449
Other determined or undetermined cause	57 (21.0)	14 (23.0)	43 (20.5)	0.541
Hemorrhagic stroke	66 (24.4)	13 (21.3)	53 (25.2)	0.328
Transient ischemic attack	33 (12.2)	5 (8.2)	28 (13.3)	0.198

WBC, white blood cell; CRP, C-reactive protein; HDL, high-density lipoprotein; LDL, low-density lipoprotein; LNT, last normal time; CT, computed tomography; IVT, Intravenous thrombolysis; EVT, endovascular thrombectomy; mRS, Modified Rankin Scale; NIHSS, National Institutes of Health Stroke Scale; and ED, emergency department.

**Table 2:** Neurological disability in patients with stroke according to short telomere length.

All	Crude Odds Ratio	95% CI	*p*-Value
mRS			
Pre-mRS	0.86	0.38–1.94	0.71
mRS at ED visit	1.26	0.71–2.24	0.42
Delta mRS	1.48	0.81–2.73	0.20
NIHSS			
NIHSS at ED visit	1.74	0.92–3.29	0.09
1 day after NIHSS	1.83	0.96–3.49	0.07
7 days after NIHSS	1.33	0.70–2.53	0.39
Acute ischemic stroke			
mRS			
mRS at ED visit	1.93	0.96–3.88	0.06
Delta mRS	2.47	1.09–5.58	0.03
NIHSS			
NIHSS at ED visit	2.66	1.31–5.44	<0.01
1 day after NIHSS	3.20	1.54–6.66	<0.01
7 days after NIHSS	1.99	0.96–4.10	0.06
Hemorrhagic stroke			
mRS			
mRS at ED visit	0.74	0.18–3.07	0.68
Delta mRS	0.52	0.13–2.13	0.36
Transient ischemic attack			
mRS			
mRS at ED visit	2.93	0.52–16.63	0.22
Delta mRS	3.16	0.64–18.87	0.18

mRS, Modified Rankin Scale; NIHSS, National Institutes of Health Stroke Scale; ED, emergency department; and CI, confidence interval.

**Table 3:** Multivariate analysis of neurological disability according to telomere length in patients with acute ischemic stroke.

	Adjusted Odds Ratio	95% CI	*p*-Value
mRS			
mRS at ED visit	3.30	0.90–12.15	0.07
Delta mRS	3.88	0.88–17.03	0.07
NIHSS			
NIHSS at ED visit	5.23	1.59–17.2	<0.01
1 day after NIHSS	7.78	1.97–30.70	<0.01
7 days after NIHSS	2.29	0.68–7.71	0.18

mRS, Modified Rankin Scale; NIHSS, National Institutes of Health Stroke Scale; and ED, emergency department.

**Table 4:** Association between short telomere length and neurological disability according to ischemic stroke subgroup.

	LAA		SVO		CE		OD or UD	
	OR (95%CI)	*p*-Value	OR (95%CI)	*p*-Value	OR (95%CI)	*p*-Value	OR (95%CI)	*p*-Value
Crude								
mRS								
At ED visit	1.01 (0.22–4.66)	0.99	0.93 (0.18–4.86)	0.94	2.86 (0.63–12.92)	0.17	2.50 (0.72–8.73)	0.15
Delta	0.85 (0.21–3.44)	0.82	1.26 (0.11–14.05)	0.85	2.51 (0.53–11.83)	0.24	8.50 (1.02–71.09)	0.05
NIHSS								
At ED visit	1.33 (0.28–6.26)	0.72	1.04 (0.22–4.96)	0.96	4.40 (0.91–21.25)	0.07	7.33 (1.76–30.61)	<0.01
After 1 day	0.93 (0.16–5.40)	0.93	3.50 (0.59–20.75)	0.17	2.86 (0.63–12.92)	0.17	6.55 (1.56–27.48)	0.01
After 7 days	0.90 (0.19–4.15)	0.89	1.63 (0.27–9.98)	0.60	1.46 (0.34–6.25)	0.61	5.14 (1.01–26.09)	0.05
Adjusted								
mRS								
At ED visit	24.29 (1.12–525.56)	0.04	0.74 (0.10–5.38)	0.77	2.62 (0.26–26.06)	0.41	2.52 (0.51–12.61)	0.26
Delta	3.37 (0.22–51.26)	0.38	6.87 (0.11–416.31)	0.36	1.14 (0.18–7.32)	0.89	13.93 (0.65–298.44)	0.09
NIHSS								
At ED visit	2.62 (0.30–22.51)	0.38	1.74 (0.07–44.36)	0.74	6.42 (0.56–73.54)	0.14	7.89 (1.32–47.25)	0.02
After 1 day	4.21 (0.29–61.68)	0.29	3.69 (0.33–40.82)	0.29	3.22 (0.29–35.46)	0.34	7.02 (1.14–43.47)	0.04
After 7 days	2.23 (0.29–17.29)	0.44	1.01 (0.11–11.05)	0.94	0.56 (0.03–10.58)	0.70	4.84 (0.67–35.02)	0.12

mRS, Modified Rankin Scale; NIHSS, National Institutes of Health Stroke Scale; ED, emergency department; LAA, large artery atherosclerosis; SVO, small vessel occlusion; CE, cardioembolism; OD, stroke of other determined etiology; UD, stroke of undetermined etiology; and CI, confidence interval; OR, odds ratio.

## Data Availability

Privacy or ethical restrictions limit the use of data.
